# The Gut of Geographically Disparate *Ciona intestinalis* Harbors a Core Microbiota

**DOI:** 10.1371/journal.pone.0093386

**Published:** 2014-04-02

**Authors:** Larry J. Dishaw, Jaime Flores-Torres, Simon Lax, Kristina Gemayel, Brittany Leigh, Daniela Melillo, M. Gail Mueller, Lenina Natale, Ivana Zucchetti, Rosaria De Santis, Maria Rosaria Pinto, Gary W. Litman, Jack A. Gilbert

**Affiliations:** 1 Division of Molecular Genetics, Department of Pediatrics, University of South Florida College of Medicine, USF Health, St. Petersburg, Florida, United States of America; 2 Division of Neonatology, University of South Florida College of Medicine, USF Health, St. Petersburg, Florida, United States of America; 3 Department of Ecology and Evolution, University of Chicago, Chicago, Illinois, United States of America; 4 Post-Baccalaureate Program in Interdisciplinary Medical Sciences, University of South Florida College of Medicine, Tampa, Florida, United States of America; 5 College of Marine Sciences, University of South Florida, St. Petersburg, Florida, United States of America; 6 Department of Animal Physiology and Evolution, Stazione Zoologica Anton Dohrn, Naples, Italy; 7 Department of Molecular Genetics, All Children's Hospital, St. Petersburg, Florida, United States of America; 8 Biosciences Division, Argonne National Laboratories, University of Chicago, Illinois, United States of America; U.S. Geological Survey, United States of America

## Abstract

It is now widely understood that all animals engage in complex interactions with bacteria (or microbes) throughout their various life stages. This ancient exchange can involve cooperation and has resulted in a wide range of evolved host-microbial interdependencies, including those observed in the gut. *Ciona intestinalis*, a filter-feeding basal chordate and classic developmental model that can be experimentally manipulated, is being employed to help define these relationships. *Ciona* larvae are first exposed internally to microbes upon the initiation of feeding in metamorphosed individuals; however, whether or not these microbes subsequently colonize the gut and whether or not *Ciona* forms relationships with specific bacteria in the gut remains unknown. In this report, we show that the *Ciona* gut not only is colonized by a complex community of bacteria, but also that samples from three geographically isolated populations reveal striking similarity in abundant operational taxonomic units (OTUs) consistent with the selection of a core community by the gut ecosystem.

## Introduction

Sequential colonization of newborns has ancient origins and has resulted in the evolution of host-symbiont interdependencies [1]–[3]. Studies comparing wildtype animals to those maintained under sterile conditions reveal bacterial influences on host development, tissue maturation and metabolic capabilities, indicating complex functional associations between host and microbe [4] that may depend on a continuous exposure to microbes [5]–[7]. Recent studies indicate that not all animals are dependent on a core microbiota. Instead, some maintain residents that fulfill core requirements (i.e., a functional core), suggesting that some interactions or interdependencies with bacteria may be less species-specific than considered previously [8], [9]. In either case, it appears that most animals can be considered metaorganisms that rely on a multitude of microbial associations [3], [4], [10]–[12]; even in marine sponges, which lack distinct body compartments, species-specific microbiota appear to be maintained [3], [13]. *Ciona intestinalis,* a sessile filter-feeding marine invertebrate chordate, possess mechanisms that likely help it regulate host-microbe interactions in the gut [14], [15]. In order to better understand and describe the composition of bacterial communities associated with the *Ciona* gut, we sampled animals from three geographically disparate populations and undertook several different approaches to define their respective gut microbiota.

## Materials and Methods

### Ethics statement

The research described herein was performed on the marine invertebrate, *Ciona intestinalis* and did not involve human or other primate subjects or mammals of any form. *Ciona* is not protected by any environmental agency in the United States (US) or in Italy. The collection services contracted in this study maintain current permits and licenses for collection and distribution of marine invertebrates to academic institutions; special permission was not required to collect these animals. Handling of live animals was in accordance with the guidelines of our academic institutions. Animals were recovered and brought to the laboratory alive and maintained in clean water with aeration. DNA was collected from the gut of dissected animals. Animal waste products were disposed of appropriately.

### Animal Collection

*Ciona intestinalis* were acquired in April 2012 from three distinct populations: near Mission Bay, San Diego (S. LePage, M-Rep, Carlsbad, CA), at the Cape Cod Canal Basin near Woods Hole (Animal Collection Services at Marine Biological Laboratories, MA), and from Fusaro Lake in Naples, Italy (Thyrrenian Sea, Italy, Animal Collection Services, Stazione Zoologica Anton Dohrn). The starved animals are designated WH(a), SD(a), and N(a) and two sets of unstarved individual animals, which were collected previously in April 2011 from the same location as WH and SD and are designated here as WH(b),(c) and SD(b),(c) (gut contents reflect diet at collection site), also are included in the analysis.

All animals were sampled immediately (at random) from the population, bagged and shipped overnight to our laboratory (in the US or Italy), and processed immediately for 16S rRNA gene PCR. Animals to be starved were maintained in 0.2 micron filtered seawater (FSW) at arrival, see below.

### Animal and sample handling

Starved animals (4–5 animals, pooled for DNA) were maintained in sterile containers at room temperature in 0.2 micron FSW (with complete water changes every 4–6 hours) for 72–96 hours to void gut content. The gut (stomach and intestine) from each animal was dissected, homogenized and total DNA was isolated (Qiagen Stool Kit) using aseptic technique and benchtop clean units. The starved samples (“a” samples), one pooled set from each of the three populations (labeled internally as Ci-121, Ci122, and Ci126 or WH, SD, and Naples, respectively), were sequenced separately as described below and used as templates to characterize the bacterial community structure. Unstarved animals, Ci-41, -42 (WH) and Ci-47, 48 (SD) were processed in the same manner, and designated in the text as samples “b” and “c,” respectively, for each population.

### 16SrRNA PCR Clone Library and Sequencing

PCR products were generated with 27F and 1492 primers [16]–[19] using Promega 2× Master Mix (Promega, Madison, WI, USA) under aseptic conditions. Products were cloned into TOPO-based cloning vectors (Invitrogen/Life Technologies, Carlsbad, CA, USA). Bacterial clones were screened for inserts by colony-PCR using vector-specific primers and sequenced on an ABI3730 automated sequencer (Applied Biosystems/Life Technologies, Carlsbad, CA, USA).

### 16SrRNA Library Preparation and Amplicon Sequencing Method

DNA template was amplified using the 515F/806R region of the 16S rRNA gene using primers and cycling conditions modified slightly from those outlined in Caporaso et al. 2011 [20], adapted for the Illumina HiSeq2000 and MiSeq by adding nine extra bases in the adapter region of the forward amplification primer that support paired-end sequencing. Briefly, the V4-5 region of the 16S rRNA gene was amplified with region-specific primers that included the Illumina flowcell adapter sequences. The reverse amplification primer also contained a twelve base barcode sequence that supports pooling of up to 2,167 different samples in each lane. PCR reactions were conducted in triplicate and products were pooled. Each pool was then quantified using PicoGreen (Invitrogen) and a plate reader. Once quantified, different volumes of each of the products were pooled into a single tube so the pool consists of an equal amount of each amplicon and cleaned using the UltraClean® PCR Clean-Up Kit (MoBIO, Carlsbad, CA, USA). Amplicons then were sequenced in a 151 bp×12 bp×151 bp MiSeq run employing custom sequencing primers and procedures described in the supplementary methods of Caporaso et al. 2012 [21].

### Sequence Analysis

All sequence analyses were completed in QIIME 1.6.0-dev [22], [23]. OTUs were selected based on 97% sequence similarity and taxonomic data were assigned to each representative sequence through the classification algorithm of the Ribosomal Database Project (RDP). Chloroplast sequences derived from eukaryotic endosymbionts were discarded, and sample libraries were rarified to a common depth of 40,528 reads before comparison. All cluster analyses are based on weighted unifrac distance because all samples were found to be dominated by a small number of high abundance OTUs; the extent to which those OTUs dominate the samples therefore is more relevant than weighting low abundance taxa disproportionality by comparing samples based on the presence or absence of OTUs.

Illumina-derived core OTUs and clone library, Sanger-sequenced, OTUs were aligned using Geneious version 6.1.6 (available from Biomatters, www.geneious.com). OTUs were compared by phylogenetic reconstruction using the Minimum Evolution method in Mega 5.2.2 [24] with 1000 bootstrap replicates and the Maximum Composite Likelihood nucleotide substitution model, to include both transitions and transversions of non-protein coding DNA sequences. Uniform rates were maintained, gaps were treated by complete deletion, and trees were inferred by Close-Neighbor-Interchange.

## Results and Discussion

### Evidence for core OTUs in the Ciona gut

**Figure 1:**
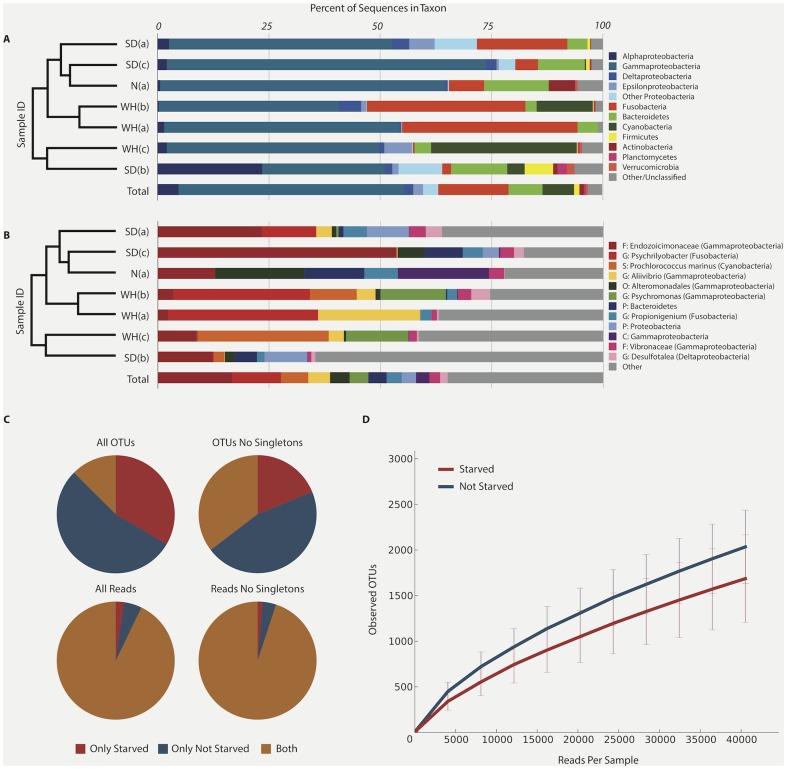
Taxonomic summary of *Ciona* samples by Illumina sequencing of 16S rRNA (alpha diversity). (**A**) Relative abundances of sequences classified to phylum, with Proteobacteria split by class. Only phyla containing at least 1% of reads across all samples are designated by color. (**B**) Relative abundances of the 12 OTUs containing at least 2% of all reads across samples. OTUs are classified to the finest taxonomic level possible, with class indicated in parentheses where appropriate. The dendrograms at left of the figures depict UPGMA clustering of the seven samples based on weighted community similarity. (**C**) Distribution of OTUs and their assigned sequences between starved and unstarved samples. Although a relatively small percent of all detected OTUs were found in both starved and unstarved samples, those OTUs comprised the vast majority of observed 16S reads, especially after discounting singleton OTUs. (**D**) Rarefaction curves for starved and unstarved communities, depicting the greater number of observed species in samples which were not starved than in those that were. Error bars are standard deviation.

Each sample was sequenced to a depth of 40,528 reads (data is deposited in MG-RAST under the following IDs: 4539627.3, 4539628.3, 4539629.3, 4539630.3, 4539624.3, 4539625.3, 4539626.3), which collectively encompassed over 9,700 unique OTUs dominated by 8 bacterial phyla (Figure 1A, 1D). Despite this high degree of community complexity, the 12 most abundant OTUs in the study were sufficient to capture at least half of the microbial diversity in all but one sample, suggesting the presence of a core gut community (Figure 1B). These 12 OTUs, 9 of which were detected in all 7 samples and all of which were detected in at least 5, each contained at least 2% of all reads in the study. Although a relatively small percentage of all detected OTUs were found in both starved and unstarved samples, those OTUs comprised the vast majority of observed 16S reads, especially after discounting singleton OTUs (OTUs containing only a single sequence across all samples) (Figure 1C). Accordingly, observed differences in community composition are largely attributable to very low abundance OTUs, and may be an artifact of limited sampling depth. Rarefaction curves depict slightly greater observed diversity in the samples taken from unstarved *Ciona* relative to their starved counterparts, although the difference is not statistically significant (Figure 1D). Overall, we found 35 OTUs shared among all 7 samples and 200 OTUs present in a least 75% (5) of the samples (Table 1 and Table S1).

**Table 1:** Core OTUs (35) found in all samples* from three geographically disparate populations.

OTU ID	N(a)	WH(a)	SD(a)	WH(b)	SD(b)	WH(c)	SD(c)	% Total	Phylum	Family
8443	5219	965	9503	1467	5028	3545	21687	16.7%	Proteobacteria	Endozoicimonaceae
8554	2	13633	4939	12420	81	124	38	11.0%	Fusobacteria	Fusobacteriaceae
8942	14	9300	1417	1685	38	1388	23	4.9%	Proteobacteria	Vibrionaceae
6994	8147	7	404	451	872	199	2402	4.4%	Proteobacteria	Unresolved (Alteromonadales)
10014	2	28	196	5974	3	5630	1	4.2%	Proteobacteria	Psychromonadaceae
1955	3043	1022	2161	906	680	38	1840	3.4%	Fusobacteria	Fusobacteriaceae
717	8324	2	27	64	23	59	133	3.0%	Proteobacteria	Unresolved (Gamma-)
2967	1396	468	1496	1219	324	735	1271	2.4%	Proteobacteria	Vibrionaceae
4938	34	174	1450	1684	352	156	874	1.7%	Proteobacteria	Desulfobulbaceae
3583	5	3	40	1	3180	12	50	1.2%	Proteobacteria	Unresolved (Alpha-)
6378	1	26	1286	172	250	1413	84	1.1%	Proteobacteria	Campylobacteraceae
9665	2	1864	276	596	16	269	20	1.1%	Proteobacteria	Shewanellaceae
9259	5	898	81	245	665	172	92	0.8%	Bacteroidetes	Unresolved
234	1	572	290	2	873	115	81	0.7%	Bacteroidetes	Flavobacteriaceae
5491	1	257	1242	2	53	141	37	0.6%	Proteobacteria	Oceanospirillaceae
1617	293	327	79	239	114	257	49	0.5%	Proteobacteria	Vibrionaceae
838	253	17	86	74	96	277	30	0.3%	Bacteroidetes	Flavobacteriaceae
1263	20	27	8	2	592	16	40	0.2%	Proteobacteria	Rhodobacteraceae
2485	12	14	67	6	467	58	68	0.2%	Proteobacteria	Rhodobacteraceae
4282	17	265	34	4	59	230	63	0.2%	Proteobacteria	Pseudoalteromonadaceae
1329	1	5	83	2	241	1	95	0.2%	Bacteroidetes	Unresolved
8677	3	2	3	12	295	57	33	0.1%	Proteobacteria	Rhodobacteraceae
6190	2	6	95	83	46	163	9	0.1%	Bacteroidetes	Campylobacteraceae
1489	5	12	98	15	34	32	39	0.1%	Proteobacteria	Alteromonadaceae
6952	11	7	38	4	108	20	31	0.1%	Proteobacteria	Rhodobacteraceae
3698	102	5	8	25	3	33	14	0.1%	Proteobacteria	Unresolved (Gamma-)
10193	8	11	1	26	79	38	17	0.1%	Proteobacteria	Desulfovibrionaceae
3473	36	12	28	6	2	8	57	0.1%	Proteobacteria	Endozoicimonaceae
6801	2	7	7	1	101	5	17	0.0%	Proteobacteria	Rhodobacteraceae
2007	42	6	24	9	2	13	40	0.0%	Proteobacteria	Endozoicimonaceae
5020	3	1	32	6	33	49	1	0.0%	Verrucomicrobia	Verrucomicrobiaceae
1910	3	3	8	6	59	20	11	0.0%	Proteobacteria	Rhodobacteraceae
6387	12	8	32	14	5	20	15	0.0%	Proteobacteria	Endozoicimonaceae
6815	2	1	1	30	8	43	3	0.0%	Proteobacteria	Unresolved (Gamma-)
8480	4	3	4	2	5	3	9	0.0%	Proteobacteria	Unresolved (Alteromonadales)

*Includes starved and unstarved animal samples. Numbers indicate the number of hits or abundance of each OTU (40,528 reads/sample). The first 9 OTUs in bold represent core OTUs that are part of the 12 most abundant OTUs described in Figure 1. Full taxonomic lineage is defined for each OTU, where possible, in Supplemental Table 1.

The gut microbiota of *Ciona* is predominated by Gram-negative bacteria and among the core OTUs (Table 1), Proteobacteria were the most abundant (28 OTUs or 80%), followed by Bacteroidetes (4 OTUs), Fusobacteria (2 OTUs), and Verrucomicrobia (1 OTU). Together, this assemblage represents 13 families of bacteria (Table 2), likely conferring broad metabolic potential to the host gut [25], [26]. In addition, OTUs related to the Gram-positive Firmicutes and Actinobacteria are likely important members of the core community and contribute several abundant OTUs in more than 75% of the samples. As evidenced by UPGMA clustering of community similarity (Figure 1A) and network clustering of sample OTUs (data not shown), there is no observed correlation either between the geographic sampling location or the sampling method (starvation) and the beta diversity relationships between samples (Table S2) as indicated by the G-test of independence producing a p value of 1 [23]. This G-test result indicates that despite whatever apparent clustering exists, two samples from the same location are not more likely to share an OTU than are samples from different locations. Similar findings were noted for starved versus unstarved animals. The detection of identical OTUs in the guts of *Ciona* from three geographically disparate populations is consistent with a global marine microbial seedbank [27].Taken together, these observations support the notion of a core microbial community in the *Ciona* gut, as shared OTUs between distant populations are sufficient to mask the effects of their highly variable environments.

**Table 2:** *Ciona* core OTUs represent four phyla, 10 orders, and 13 families of bacteria.

Core Phyla	Order	Family	OTUs
Bacteroidetes	Flavobacteriales	Flavobacteriaceae	2
	*		2
Fusobacteria	Fusobacteriales	Fusobacteriaceae	2
Proteobacteria	Alteromonadales	Pseudoalteromonadaceae	1
		Psychromonadaceae	1
		Shewanella	1
	Campylobacterales	Campylobacteraceae	2
	Desulfobacterales	Desulfobulbaceae	1
	Desulfovibronales	Desulfovibrionaceae	1
	Oceanospirillales	Hahellaceae	4
		Oceanospirillaceae	1
	Rhodobacterales	Rhodobacteraceae	6
	Vibrionales	Vibrionaceae	4
	*		6
Verrucomicrobia	Verrucomicrobiales	Rubritalea	1
			35

*OTUs that cannot be taxonomically sorted below class.

### The Ciona gut microbiota supports several familiar OTUs

The observed differences in core OTUs between starved and unstarved *Ciona* samples (Figure 1; Figure S1; Table S1 and S2) suggest that some core OTUs represent planktonic communities (e.g., *Prochlorococcus*) while others potentially form adherent communities in stable biofilms within the mucus layers (e.g., anaerobic *Psychrilyobacter* sp.). It is possible that adherent communities benefit from early colonization of the gut surface at the onset of feeding and are influenced by host responses. Thus, colonization is likely influenced by physical properties of the *Ciona* gut (e.g., the presence of unique side chains in mucin-like glycoproteins [28]) as well as the types of bacteria colonizing planktonic or dietary particles.

It is likely that some members of this microbiota, such as anaerobic Fusobacteria, stably associate with distinct compartments of the *Ciona* gut, and together with other members of the gut microbiota influence host fitness. Some surfaces of marine invertebrates can be colonized by specific groups of bacteria, such as members of the Oceanospirillales order [29]–[33]. In the *Ciona* gut, OTUs closely related (>97% identity) to *Endozoicomonas* sp., members of Oceanospirillales, can peak at 53.5% relative abundance (Table S1). *Endozoicomonas* sp. may serve essential roles in some marine invertebrates as they can dominate the surface mucus of many corals [31]–[35], be associated with internal tissue spaces [29], and be endosymbionts [36], [37]. Diverse invertebrates appear to associate closely with these bacteria [30], [38], which are predicted to serve important roles in metabolizing complex organic compounds [39] and even in sulfur recycling [40], similar to other members of the Order Oceanospirillales. However, it remains unclear if they are found as part of other marine invertebrate microbiomes [3], [41], [42].

Overall, the abundance of core OTUs in *Ciona* can vary considerably. The abundance of a *Psychrilyobacter* (Fusobacteria)-related OTU, for example, can reach 30% in some samples. In addition, the abundance of several other OTUs within Bacteroidetes and Gammaproteobacteria can vary in the *Ciona* gut even among starved animals (Table S1). Bacterial communities that associate with some coastal invertebrates are subject to anthropomorphic influences [31], [32]. We surveyed marine invertebrate-associated microbial communities by screening 16S sequences in the available databases and recorded OTU matches (>97% identity) in various animal taxa (Table S3). More specifically, *Ciona* core OTUs were compared to coral gastric cavity [43], polychaete intestine [42], abalone intestine, and two sea cucumber intestinal microbiomes [44], [45] and the sharing of some bacterial families and genera were noted (Table 3). However, this sharing was not across any significant number of genera, with the exception of one sea cucumber, which shared 12 OTUs among 5 unique genera with *Ciona* (Table 3). About 29% of the *Ciona* core OTUs (10 of 35) have been identified in the gut of various sea cucumbers species (although not all in any one species; Table S3). Indeed, recent work has revealed that many invertebrates maintain bacterial communities of related (upper-level) taxonomic groups composed of shared classes and families within Proteobacteria, Fusobacteria, Bacteroidetes, Actinobacteria, and Firmicutes in their guts. Whereas a few of the *Ciona* core OTUs previously have only been described in environmental samples (Table S3), most of the core OTUs have been described as associated with other animal taxa, for example, the Fusobacteria genera, *Psychrilyobacter* and *Propionigenium*. Taken together, these findings suggest that some bacterial families form abundant communities in the gut environment of diverse marine invertebrates, possibly demonstrating a preference for the feeding habits and/or the gut ecosystem of these animals.

**Table 3:** Comparison of the 35 *Ciona* core gut OTUs to other marine invertebrate gut microbiota.

Subject organism^1^	Gut OTUs reported	Phyla reported^2^	Phylum-level hits^3^	Family-level hits^3^	Genus-level hits^3^	Unique Genus hits
Coral (*Galaxea fascicularis*) gastric cavity	17	3	11	6	4	1^a^
Polychaete (*Neanthes glandicincta*) intestine	84	6	32	15	5	1^b^
Abalone (*Haliotis discuss hannai*) intestine^4^	63	5	56	50	36	2^c^
Sea cucumber (*Holothuria leucospilota*) intestine	139	3	53	48	48	2^d^
Sea cucumber (*Apostichopus japonicus*) intestine	217	8	131	110	12	5^e^

All comparisons are between *Ciona* gut core OTUs and other marine invertebrates. Note that priming strategies and sequencing methods vary greatly. Therefore, OTU similarities are approximate however at >95% confidence over the regions compared. ^1^Number of OTUs sharing *taxonomic* hits (with Ciona core OTUs) in BLAST database searches. ^1^References cited in the text. ^2^Unclassified bacteria not shown. ^3^Number of subject OTUs shared with *Ciona* core OTUs at each taxonomic level specified. ^4^Unpublished data deposited in NCBI. *^a^Endozoicomonas; ^b^Vibrio; ^c^Vibrio* and *Shewanella; ^d^Shewanella* and *Pseudoalteromonas; ^e^Vibrio, Rubritalea, Arcobacter, Pseudoalteromonas, and Shewanella.*

### Illumina sequencing reaffirms OTU findings from clone library surveys

**Figure 2:**
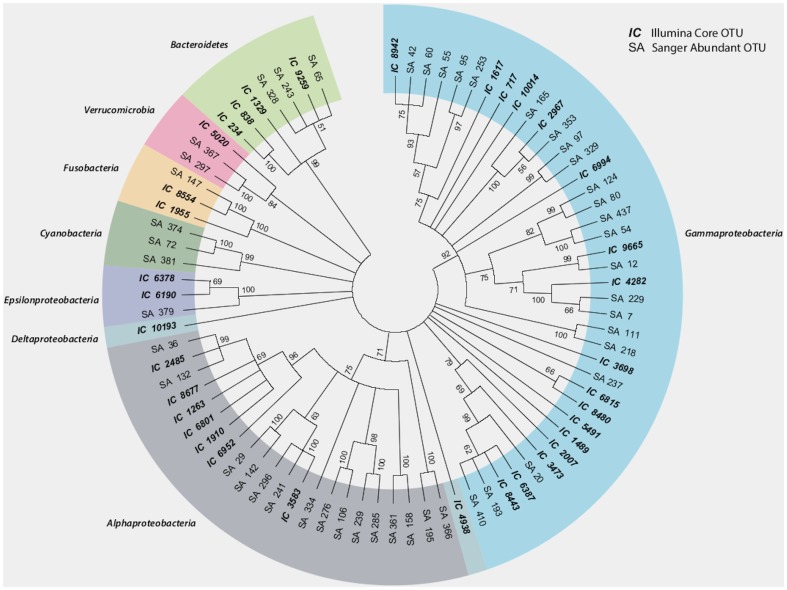
Unrooted distance tree comparing Illumina-derived core OTUs and abundant Sanger-sequenced clone library 16S samples. Pairwise comparisons of OTUs indicates that a significant proportion of the core OTUs were captured consistently over a three year collection period spanning all seasons.

Although the power of the studies reported here is somewhat limited by the sample size, it is noted that between 2010-12, 16S rRNA PCR clone library surveys of 70 animals (46 from WH, 20 from SD, and 4 from Naples) also were conducted. Samples were collected randomly throughout each year; some were starved, others had full guts and others had guts devoid of fecal matter. Approximately 3400 Sanger-based sequencing reads (nearly 1700 clones sequenced in both directions) resulted in 1617 near full-length 16S sequences (Genbank accession KF798359-KF799975) resulting in 442 unique OTUs. The 47 most abundant OTUs (seen more than >5 times) are markedly similar to the core community that is predicted by Illumina sequencing of the starved and unstarved sample sets (Figure 2; Table S4; Table S5). This finding reaffirms that the core bacterial community within the *Ciona* gut is stably associated, abundant, and can be captured irrespective of handling or sampling method.

In interpreting host-OTU relationships between collection sites, it should be noted that recent evidence indicates *Ciona intestinalis* could be experiencing a speciation event currently defined by two subtypes [46]–[49]. The ‘A’ subtype is represented by populations in the Mediterranean and the west coast of North America (here Naples and SD, respectively); the ‘B’ subtype is found along Scandinavian coastlines and along the northeastern coast of North America (represented here by WH). Both subtypes are geographically restricted and demonstrate distinct ranges of temperature tolerance. Recently, a unique assortment of microbiota inhabiting the tunic of *Ciona intestinalis* was described from ‘B’ subtype animals (WH samples) [50]. Notably, almost no overlap (i.e., shared OTUs) exists with the microbiota of the gut.

### Selection of a core microbiota may not be limited to feeding habits and environment

A complex assemblage of a stably associated core bacterial community has been defined in the gut of *Ciona intestinalis*, sampled from three disparate geographic locations. To our knowledge, this is the first report of a core gut microbiota from a marine invertebrate under starved and unstarved conditions. *Ciona* may have evolved some level of symbiotic interdependences, possibly extending the range of host physiological responses, which could be targeted to identify symbiotic interactions across the gut epithelium. Studies of the host-microbial dialogue of the gut could help reveal basic requirements that drive and/or help sustain immune homeostasis of the gut. Immune-type molecules, such as the immunoglobulin variable region-containing chitin binding proteins (VCBPs), are expressed in high abundance in the gut epithelium and it remains to be determined what role, if any, these molecules play in modulating the composition of closely-associated gut microbial communities. VCBPs are secreted proteins that have been shown to bind bacteria both *in vivo* and *in vitro;* because they associate closely with epithelial processes and microvilli in the gut lumen [15], it is most likely that they interact with bacteria adhering to the gut mucosa. Determining the precise mechanisms by which *Ciona* establishes and maintains its gut microbial ecosystem, from both anatomical and immunological perspectives, could have important implications for our understanding of the evolution of gut immunity and the nature of innate immunity at the gut epithelial interface [14].

## Supporting Information

Figure S1 **Summary of the number of core OTUs shared between all (left), not starved (center), and starved (right) samples.** Bins are the number of samples necessary for an OTU to be detected and be considered “core” and the height is the number of OTUs in that bin.
(PDF)

Table S1 **Complete OTU spreadsheet with data on four sheets: OTU_Table_ALL, sorted OTU_Table_ALL, Abundant_OTUs, and core OTUs in all 7 samples.** NOTE: Starved samples are designated as (a) and unstarved samples are (b) and (c).
(ZIP)

Table S2 **Pairwise sample comparisons and beta diversity calculations.**
(ZIP)

Table S3 **Taxonomic organization and classification of Ciona intestinalis gut core OTUs (35).**
(ZIP)

Table S4 **Finest Taxonomic Classification Comparing Clone Library OTUs and Illumina Sequencing-Derived “Core OTUs” using Sequence Match from RDP at 95% Threshold. Database screening, Dec 2013.**
(ZIP)

Table S5 ***Ciona*** **core OTUs and clone library-derived Sanger-sequenced OTUs for download.**
(ZIP)

## References

[1] Franzenburg S, Fraune S, Altrock PM, Kunzel S, Baines JF, et al (2013) Bacterial colonization of Hydra hatchlings follows a robust temporal pattern. ISME J 7: 781–790. 23344242 10.1038/ismej.2012.156 PMC3603398

[2] Mao-Jones J, Ritchie KB, Jones LE, Ellner SP (2010) How microbial community composition regulates coral disease development. PLoS Biol 8: e1000345. 20361023 10.1371/journal.pbio.1000345 PMC2846858

[3] Schmitt S, Tsai P, Bell J, Fromont J, Ilan M, et al (2012) Assessing the complex sponge microbiota: core, variable and species-specific bacterial communities in marine sponges. ISME J 6: 564–576. 21993395 10.1038/ismej.2011.116 PMC3280146

[4] McFall-Ngai M, Hadfield MG, Bosch TC, Carey HV, Domazet-Loso T, et al (2013) Animals in a bacterial world, a new imperative for the life sciences. Proc Natl Acad Sci U S A 110: 3229–3236. 23391737 10.1073/pnas.1218525110 PMC3587249

[5] Chung H, Pamp SJ, Hill JA, Surana NK, Edelman SM, et al (2012) Gut immune maturation depends on colonization with a host-specific microbiota. Cell 149: 1578–1593. 22726443 10.1016/j.cell.2012.04.037 PMC3442780

[6] Rhee KJ, Sethupathi P, Driks A, Lanning DK, Knight KL (2004) Role of commensal bacteria in development of gut-associated lymphoid tissues and preimmune antibody repertoire. J Immunol 172: 1118–1124. 14707086 10.4049/jimmunol.172.2.1118

[7] McFall-Ngai MJ (2002) Unseen forces: the influence of bacteria on animal development. Dev Biol 242: 1–14. 11795936 10.1006/dbio.2001.0522

[8] Sullam KE, Essinger SD, Lozupone CA, O'Connor MP, Rosen GL, et al (2012) Environmental and ecological factors that shape the gut bacterial communities of fish: a meta-analysis. Mol Ecol 21: 3363–3378. 22486918 10.1111/j.1365-294X.2012.05552.x PMC3882143

[9] Yatsunenko T, Rey FE, Manary MJ, Trehan I, Dominguez-Bello MG, et al (2012) Human gut microbiome viewed across age and geography. Nature 486: 222–227. 22699611 10.1038/nature11053 PMC3376388

[10] Gordon JI (2012) Honor thy gut symbionts redux. Science 336: 1251–1253. 22674326 10.1126/science.1224686

[11] Xu J, Gordon JI (2003) Honor thy symbionts. Proc Natl Acad Sci U S A 100: 10452–10459. 12923294 10.1073/pnas.1734063100 PMC193582

[12] Plante CJ, Jumars PA, Baross JA (1990) Digestive Associations Between Marine Detritovores and Bacteria. Ann Rev Ecol Syst 21: 93–127.

[13] Fan L, Reynolds D, Liu M, Stark M, Kjelleberg S, et al (2012) Functional equivalence and evolutionary convergence in complex communities of microbial sponge symbionts. Proc Natl Acad Sci U S A 109: E1878–1887. 22699508 10.1073/pnas.1203287109 PMC3390844

[14] Dishaw LJ, Flores-Torres JA, Mueller MG, Karrer CR, Skapura DP, et al (2012) A basal chordate model for studies of gut microbial immune interactions. Front Immunol 3: 96. 22563328 10.3389/fimmu.2012.00096 PMC3342567

[15] Dishaw LJ, Giacomelli S, Melillo D, Zucchetti I, Haire RN, et al (2011) A role for variable region-containing chitin-binding proteins (VCBPs) in host gut-bacteria interactions. Proc Natl Acad Sci U S A 108: 16747–16752. 21930927 10.1073/pnas.1109687108 PMC3189038

[16] Weisburg WG, Barns SM, Pelletier DA, Lane DJ (1991) 16S ribosomal DNA amplification for phylogenetic study. J Bacteriol 173: 697–703. 1987160 10.1128/jb.173.2.697-703.1991 PMC207061

[17] Lane DJ (1991) rRNA Sequencing. In: Stackebrandt E, Goodfellow M, editors. Nucleic Acid Techniques in Bacterial Systematics. New York: John Wiley & Sons. pp. 115–147.

[18] Ibrahim A, Goebel BM, Liesack W, Griffiths M, Stackebrandt E (1993) The phylogeny of the genus Yersinia based on 16S rDNA sequences. FEMS Microbiol Lett 114: 173–177. 7506685 10.1111/j.1574-6968.1993.tb06569.x

[19] Stackebrandt E, Liesack W (1993) Handbook of new bacterial systematics. In: Goodfellow M, O'Donnell AG, editors. Handbook of New Bacterial Systematics. London: Academic Press. pp. 152–189.

[20] Caporaso JG, Lauber CL, Walters WA, Berg-Lyons D, Lozupone CA, et al (2011) Global patterns of 16S rRNA diversity at a depth of millions of sequences per sample. Proc Natl Acad Sci U S A 108 Suppl 14516–4522. 20534432 10.1073/pnas.1000080107 PMC3063599

[21] Caporaso JG, Paszkiewicz K, Field D, Knight R, Gilbert JA (2012) The Western English Channel contains a persistent microbial seed bank. ISME J 6: 1089–1093. 22071345 10.1038/ismej.2011.162 PMC3358019

[22] Caporaso JG, Kuczynski J, Stombaugh J, Bittinger K, Bushman FD, et al (2010) QIIME allows analysis of high-throughput community sequencing data. Nat Methods 7: 335–336. 20383131 10.1038/nmeth.f.303 PMC3156573

[23] Kuczynski J, Stombaugh J, Walters WA, Gonzalez A, Caporaso JG, et al.. (2012) Using QIIME to analyze 16S rRNA gene sequences from microbial communities. Curr Protoc Microbiol Chapter 1: Unit 1E 5. 10.1002/9780471729259.mc01e05s27 PMC4477843 23184592

[24] Tamura K, Peterson D, Peterson N, Stecher G, Nei M, et al (2011) MEGA5: molecular evolutionary genetics analysis using maximum likelihood, evolutionary distance, and maximum parsimony methods. Mol Biol Evol 28: 2731–2739. 21546353 10.1093/molbev/msr121 PMC3203626

[25] Savage DC (1977) Microbial ecology of the gastrointestinal tract. Annu Rev Microbiol 31: 107–133. 334036 10.1146/annurev.mi.31.100177.000543

[26] Ley RE, Lozupone CA, Hamady M, Knight R, Gordon JI (2008) Worlds within worlds: evolution of the vertebrate gut microbiota. Nat Rev Microbiol 6: 776–788. 18794915 10.1038/nrmicro1978 PMC2664199

[27] Gibbons SM, Caporaso JG, Pirrung M, Field D, Knight R, et al (2013) Evidence for a persistent microbial seed bank throughout the global ocean. Proc Natl Acad Sci U S A 110: 4651–4655. 23487761 10.1073/pnas.1217767110 PMC3607043

[28] Johansson ME, Larsson JM, Hansson GC (2011) The two mucus layers of colon are organized by the MUC2 mucin, whereas the outer layer is a legislator of host-microbial interactions. Proc Natl Acad Sci U S A 108 Suppl 14659–4665. 20615996 10.1073/pnas.1006451107 PMC3063600

[29] Bayer T, Neave MJ, Alsheikh-Hussain A, Aranda M, Yum LK, et al (2013) The microbiome of the Red Sea coral Stylophora pistillata is dominated by tissue-associated endozoicomonas bacteria. Appl Environ Microbiol 79: 4759–4762. 23709513 10.1128/AEM.00695-13 PMC3719505

[30] Kurahashi M, Yokota A (2007) Endozoicomonas elysicola gen. nov., sp. nov., a gamma-proteobacterium isolated from the sea slug Elysia ornata. Syst Appl Microbiol 30: 202–206. 16904280 10.1016/j.syapm.2006.07.003

[31] Morrow KM, Moss AG, Chadwick NE, Liles MR (2012) Bacterial associates of two Caribbean coral species reveal species-specific distribution and geographic variability. Appl Environ Microbiol 78: 6438–6449. 22773636 10.1128/AEM.01162-12 PMC3426691

[32] Vezzulli L, Pezzati E, Huete-Stauffer C, Pruzzo C, Cerrano C (2013) 16SrDNA Pyrosequencing of the Mediterranean Gorgonian Reveals a Link among Alterations in Bacterial Holobiont Members, Anthropogenic Influence and Disease Outbreaks. PLoS One 8: e67745. 23840768 10.1371/journal.pone.0067745 PMC3694090

[33] Bayer T, Arif C, Ferrier-Pages C, Zoccola D, Aranda M, et al (2013) Bacteria of the genus Endozoicomonas dominate the microbiome of the Mediterranean gorgonian coral Eunicella cavolini. Mar Ecol Prog Ser 479: 75–84.

[34] Correa H, Haltli B, Duque C, Kerr R (2013) Bacterial communities of the gorgonian octocoral Pseudopterogorgia elisabethae. Microb Ecol 66: 972–985. 23913197 10.1007/s00248-013-0267-3

[35] Carlos C, Torres TT, Ottoboni LM (2013) Bacterial communities and species-specific associations with the mucus of Brazilian coral species. Sci Rep 3: 1624. 23567936 10.1038/srep01624 PMC3620669

[36] Jensen S, Duperron S, Birkeland NK, Hovland M (2010) Intracellular Oceanospirillales bacteria inhabit gills of Acesta bivalves. FEMS Microbiol Ecol 74: 523–533. 21044098 10.1111/j.1574-6941.2010.00981.x

[37] Zielinski FU, Pernthaler A, Duperron S, Raggi L, Giere O, et al (2009) Widespread occurrence of an intranuclear bacterial parasite in vent and seep bathymodiolin mussels. Environ Microbiol 11: 1150–1167. 19226299 10.1111/j.1462-2920.2008.01847.x

[38] Goffredi SK, Johnson SB, Vrijenhoek RC (2007) Genetic diversity and potential function of microbial symbionts associated with newly discovered species of Osedax polychaete worms. Appl Environ Microbiol 73: 2314–2323. 17277220 10.1128/AEM.01986-06 PMC1855680

[39] Brenner DJ, Krieg NR, Garrity GM, Staley JT (2005) Bergey's Manual of Systematic Bacteriology: The Proteobacteria. New York, N.Y.: Springer.

[40] Raina JB, Tapiolas D, Willis BL, Bourne DG (2009) Coral-associated bacteria and their role in the biogeochemical cycling of sulfur. Appl Environ Microbiol 75: 3492–3501. 19346350 10.1128/AEM.02567-08 PMC2687302

[41] King GM, Judd C, Kuske CR, Smith C (2012) Analysis of stomach and gut microbiomes of the eastern oyster (Crassostrea virginica) from coastal Louisiana, USA. PLoS One 7: e51475. 23251548 10.1371/journal.pone.0051475 PMC3520802

[42] Li M, Yang H, Gu JD (2009) Phylogenetic diversity and axial distribution of microbes in the intestinal tract of the polychaete Neanthes glandicincta. Microb Ecol 58: 892–902. 19572164 10.1007/s00248-009-9550-8

[43] Agostini S, Suzuki Y, Higuchi T, Casareto BE, Yoshinaga K, et al (2012) Biological and chemical characteristics of the coral gastric cavity. Coral Reefs 31: 147–156.

[44] Zhang X, Nakahara T, Miyazaki M, Nogi Y, Taniyama S, et al (2012) Diversity and function of aerobic culturable bacteria in the intestine of the sea cucumber Holothuria leucospilota. J Gen Appl Microbiol 58: 447–456. 23337580 10.2323/jgam.58.447

[45] Zhang X, Nakahara T, Murase S, Nakata H, Inoue T, et al (2013) Physiological characterization of aerobic culturable bacteria in the intestine of the sea cucumber Apostichopus japonicus. J Gen Appl Microbiol 59: 1–10. 23518513 10.2323/jgam.59.1

[46] Caputi L, Andreakis N, Mastrototaro F, Cirino P, Vassillo M, et al (2007) Cryptic speciation in a model invertebrate chordate. Proc Natl Acad Sci U S A 104: 9364–9369. 17517633 10.1073/pnas.0610158104 PMC1890500

[47] Roux C, Tsagkogeorga G, Bierne N, Galtier N (2013) Crossing the species barrier: genomic hotspots of introgression between two highly divergent Ciona intestinalis species. Mol Biol Evol. 10.1093/molbev/mst066 23564941

[48] Iannelli F, Pesole G, Sordino P, Gissi C (2007) Mitogenomics reveals two cryptic species in Ciona intestinalis. Trends Genet 23: 419–422. 17640763 10.1016/j.tig.2007.07.001

[49] Nydam ML, Harrison RG (2010) Polymorphism and divergence within the ascidian genus Ciona. Mol Phylogenet Evol 56: 718–726. 20403444 10.1016/j.ympev.2010.03.042

[50] Blasiak LC, Zinder SH, Buckley DH, Hill RT (2013) Bacterial diversity associated with the tunic of the model chordate Ciona intestinalis. ISME J. 10.1038/ismej.2013.156 PMC3906817 24048225

