# Supplementary figures and images for: The Gut of Geographically Disparate Ciona intestinalis Harbors a Core Microbiota
Source: PLoS One. 2014 Apr 2;9(4):e93386. doi: 10.1371/journal.pone.0093386 (PMC3973685; doi:10.1371/journal.pone.0093386)

Number of Shared OTUs

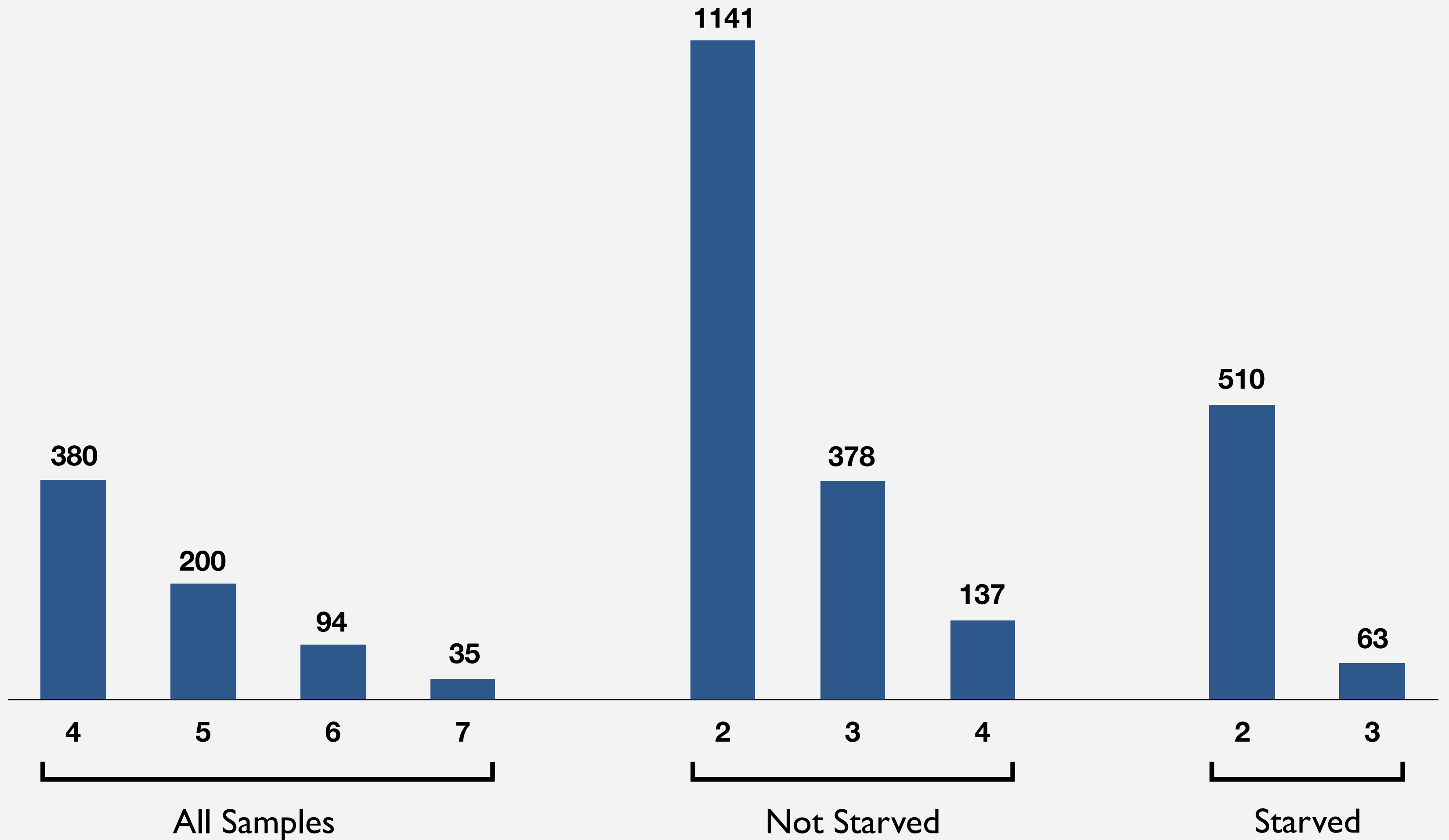

Number of Samples Containing Shared OTU

Supplement: Figure S1 — Summary of the number of core OTUs shared between all (left), not starved (center), and starved (right) samples. Bins are the number of samples necessary for an OTU to be detected and be considered “core” and the height is the number of OTUs in that bin. (PDF) [file pone.0093386.s001.pdf]
